# Metropolitan Medical Schools and Their Common Interests

**Published:** 1893-09-16

**Authors:** 


### The Hospital

An Institutional Journal of

Science, Medicine, Nursing, and Philanthropy.

*For Hospitals, Asylums, Infirmaries, Dispensaries, Medical Practitioners, Students Nurses, and the Charitable Public.*

Vol. XIV.—No. 364.] [Sept. 16, 1893.

### METROPOLITAN MEDICAL SCHOOLS AND THEIR COMMON INTERESTS.

In spite of defects, many of which might easily be removed, the system of medical training that prevails in London has yielded results that fully entitle the greatest city in the world to rank as one of the chief centres of medical education. The most essential fact about the London medical schools is that they are hospital schools. School and hospital are incorporate—they are under the same management. The teachers in the school and the staff of the hospital are appointed by the same body, and all the regulations as to the conditions under which clinical teaching is to be imparted in the hospital are made by governors who have the welfare of the school closely at heart. Therein the London schools differ from the Universities and most provincial schools, where the authorities at hospital and medical school form bodies quite distinct, and where the admission of students to the wards is in terms of a contract between these governing bodies. From the students' point of view the advantages of the London system in so far as clinical teaching is concerned are not to be exaggerated. The London medical schools owe more than three-fourths of their success to their association with London hospitals. It was stated by Sir Andrew Clark before the Lords Committee on Metropolitan Hospitals that "the medical education in London is about the most practical education given anywhere in the world." It is to give the highest praise to the London system to say that with regard to the medical man who has been educated at a London school it is generally safe to infer that he has seen much of actual disease, that he is conversant with all the problems that arise at the bedside, that, in short, he is a thoroughly practical doctor.

Although the London system is excellent it is not perfect. It has drawbacks, many of which might be removed if the several schools would co-operate a little more than they do. There are eleven medical schools in London, and there is keen rivalry between them. This is but natural, and in many directions the result is good. The striving for greater efficiency to which the competition for the favour of the public gives rise is an advantage in every way from the students' standpoint. But the rivalry should be wisely directed, and it should not prevent co-operation where co-operation is called for. There are two points as to which it seems pretty clear that the schools might join hands without loss to themselves, and with distinct advantage to medical education in London.

Of the eleven London schools ten announce that they make full provision for preparing students not only for the examinations of the conjoint colleges, but for those of the University of London also. In order to enter for the London M.B., one must have passed what is called the preliminary scientific (M.B.) examination of the University. To obtain a bare pass, not to speak of honours, one must have a good elementary knowledge of chemistry, physics, and general biology. At all the London schools except Middlesex, special classes are provided in these subjects. Middlesex refers those who desire preliminary instruction in science to University College or King's College. When the student has passed the preliminary scientific he comes back to Middlesex to receive a very thorough instruction in those subjects on which the intermediate and final examinations of London University are based. The example set by Middlesex is one that deserves to be followed. That each school should have special classes in subjects that belong to a science not to a medical curriculum, and that are better taught at a science school, constitutes a waste of time, energy, and money that is to be regretted, all the more so in view of the fact that at most of the schools, the percentage of students who go forward for the London University degrees is small. Of course, there are difficulties in the way. The larger schools have made provision for these classes on so large a scale that they cannot afford to give them up, even although they desired to do; and the smaller schools are so eager to hold their own that they will not have it said of them that they are not every whit as well equipped as the best. Moreover, when the authorities of Middlesex advise students to go to University College or King's College, they may be held to run a risk of the students not coming back. As a matter of fact, however, they do come back. This should give courage to other schools to do as Middlesex does.

Another matter as to which co-operation is to be desired is of even greater importance. It is a matter which is of special interest to those who, having completed the five years' curriculum and obtained a registrable qualification, desire to devote a year or two to clinical study. What are the opportunities open in London to a young man in this case? Unless he is of sufficient wealth to pay the fee for hospital practice at several hospitals, he must confine himself to the hospital at which he has been trained, and with the work of which he is already familiar. True, by taking the London Post Graduate course, he may procure admission to some of the special hospitals. Of these opportunities the good student will make good use, but how meagre are they in view of the unrivalled and unlimited field for clinical instruction that exists in the metropolis! What is urgently needed is what Mr. John Erichsen, in a very suggestive pamphlet issued by him last year, calls "hospital federation for clinical purposes." Mr. Erichsen, impressed with the undoubted fact that "no city in the civilised world offers such a wealth, and so varied an abundance of clinical material as does London," goes on to say, "I cannot doubt that with proper organisation of the enormous opportunities for clinical study that exist in London, and by rendering these accessible to the advanced student and young practitioner, the necessity for foreign study will no longer be felt, and that the cliniques of London will attract in their turn students, not only from all parts of the United Kingdom, but from our Colonies, the United States of America, and even the Continent of Europe." When it is remembered that Mr. Erichsen includes in his survey of metropolitan hospitals the eleven general hospitals which represent a sum total of 4,542 beds, not to speak of their enormous out-patient departments, the twenty-seven poor law infirmaries, with beds for 13,285 patients; a number of important special hospitals; and the lunatic asylums in and around London, who will say that the possibilities of the case are exaggerated?

But how is a result so desirable to be brought about? Mr. Erichsen has formulated a plan to which the only objection is that it presupposes the existence of the Teaching University of London, for which we have waited so long that we have begun to doubt whether it will come in this generation. To this Teaching University it is proposed that the several medical schools shall be admitted as constituent colleges, it being a condition of admission that each school shall become federated so far as advanced clinical hospital instruction is concerned with the other constituent medical colleges. To use Mr. Erichsen's own words: "The federated hospitals should be open for the purposes of clinical instruction to all those students who intend to present themselves for examination for the M.D. degree of the New University. . . . . Whether Mr Erichsen's plan is the best, or whether a better, independent of the annoying conditions which still deprive London of a Teaching University, can be devised—there can be no question as to the desirability of the object in view. That object deserves to have the active and united support of all who are engaged in the work of the London medical schools and of the London hospitals.

